# Variable Domain *N*-Linked Glycans Acquired During Antigen-Specific Immune Responses Can Contribute to Immunoglobulin G Antibody Stability

**DOI:** 10.3389/fimmu.2018.00740

**Published:** 2018-04-12

**Authors:** Fleur S. van de Bovenkamp, Ninotska I. L. Derksen, Mariëlle J. van Breemen, Steven W. de Taeye, Pleuni Ooijevaar-de Heer, Rogier W. Sanders, Theo Rispens

**Affiliations:** ^1^Sanquin Research, Department of Immunopathology, Amsterdam, Netherlands; ^2^Landsteiner Laboratory, Academic Medical Centre, University of Amsterdam, Amsterdam, Netherlands; ^3^Academic Medical Centre, Department of Medical Microbiology, University of Amsterdam, Amsterdam, Netherlands; ^4^Department of Microbiology and Immunology, Weill Medical College of Cornell University, New York, NY, United States

**Keywords:** antibody stability, variable domain glycosylation, Fab glycosylation, adalimumab, IVIg, thermal unfolding

## Abstract

Immunoglobulin G (IgG) can contain *N*-linked glycans in the variable domains, the so-called Fab glycans, in addition to the Fc glycans in the C_H_2 domains. These Fab glycans are acquired following introduction of *N*-glycosylation sites during somatic hypermutation and contribute to antibody diversification. We investigated whether Fab glycans may—in addition to affecting antigen binding—contribute to antibody stability. By analyzing thermal unfolding profiles of antibodies with or without Fab glycans, we demonstrate that introduction of Fab glycans can improve antibody stability. Strikingly, removal of Fab glycans naturally acquired during antigen-specific immune responses can deteriorate antibody stability, suggesting *in vivo* selection of stable, glycosylated antibodies. Collectively, our data show that variable domain *N*-linked glycans acquired during somatic hypermutation can contribute to IgG antibody stability. These findings indicate that introducing Fab glycans may represent a mechanism to improve therapeutic/diagnostic antibody stability.

## Introduction

**Figure 1:**
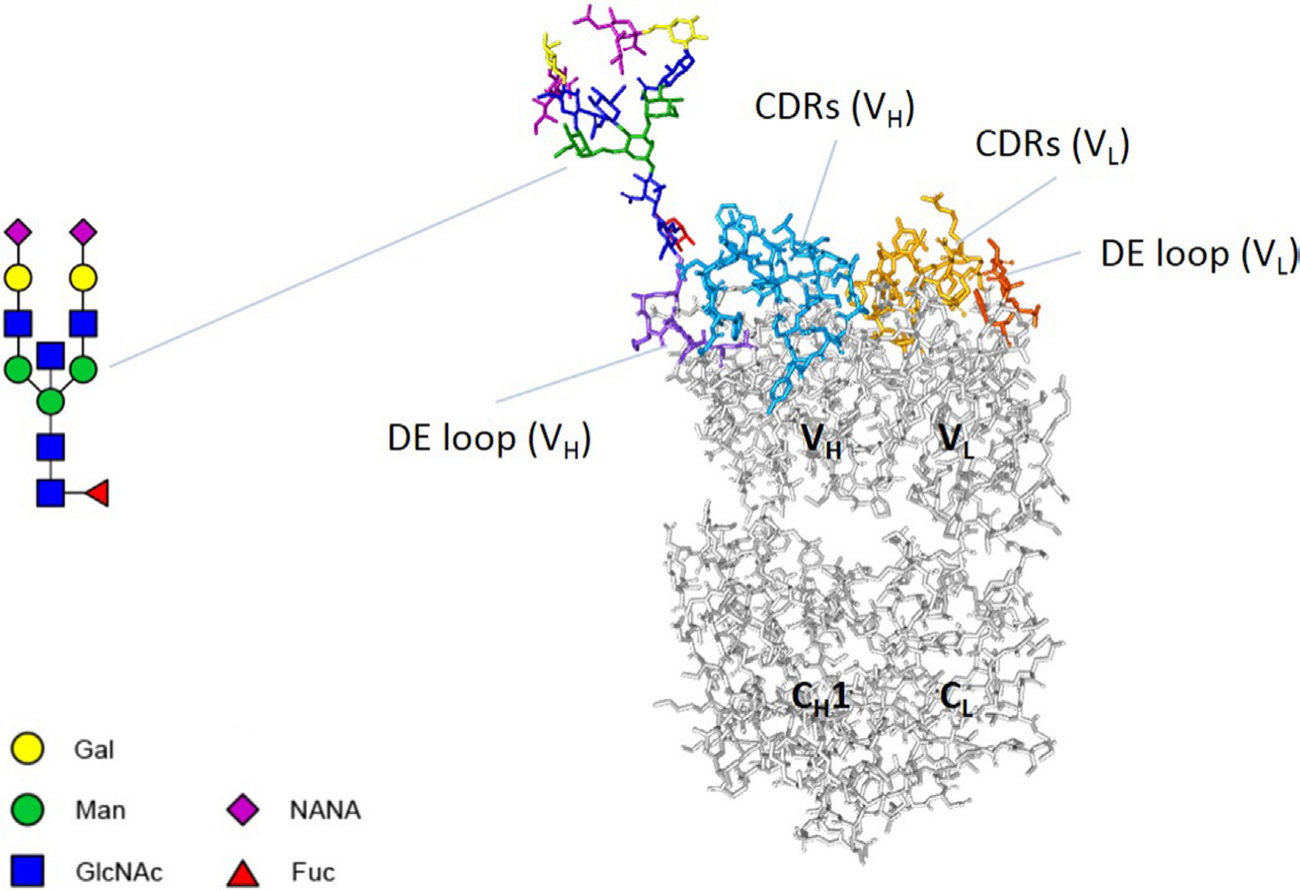
Fab glycosylation. An antibody Fab arm consists of two variable domains (VH and VL) and two constant domains (CH1 and CL). Both variable domains contain three complementarity determining regions (CDRs) and a DE loop. The Fab arm of adalimumab NH82 contains a glycan at position 82 of the heavy chain. A biantennary glycan was modeled using the online tool Sweet (http://www.glycosciences.de/modeling/sweet2/doc/index.php) (4). Subsequently, using Discovery Studio Visualizer v16.1 and UCSF Chimera v1.11.2, the glycan was introduced into the crystal structure of adalimumab Fab (PDB 3WD5), attached to N82 of the heavy chain. The side chain of N82 was reoriented to allow a structurally/chemically plausible linkage with the glycan. The resulting structure was not further optimized. Abbreviations: Gal, galactose; Man, mannose; GlcNAc, *N*-acetylglucosamine; NANA, *N*-acetylneuraminic acid; Fuc, fucose.

Immunoglobulin G (IgG) is the most abundant class of immunoglobulins in human serum. IgGs can contain *N*-linked glycans in the variable domains, the so-called Fab glycans (Figure 1), in addition to the Fc glycans in the C_H_2 domains. Fab glycans are present in about 15% of circulating IgGs (1). These Fab glycans are acquired following introduction of *N*-glycosylation sites during somatic hypermutation and thereby constitute part of the physiological repertoire of antibodies (2). We previously showed that these *N*-glycosylation sites are acquired predominantly at positions in the variable domains where a single nucleotide mutation turns a “latent” or “progenitor” glycosylation motif into an actual *N*-glycosylation motif. We furthermore showed that Fab glycans are usually localized close to antigen-binding sites and can influence antigen binding and contribute to affinity maturation (3). However, we also observed only minimal or no effects in quite a few cases, suggesting other functions of Fab glycans may exist.

It has been suggested that glycans in the variable domains enhance antibody aggregation (5–7). On the other hand, recent literature indicates that Fab glycans may contribute to antibody solubility and stability and may prevent aggregation (8–10). For example, Courtois and colleagues showed that introduction of glycans specifically introduced to mask aggregation-prone regions improves antibody stability to a similar extent as removing those regions by replacement of hydrophobic amino acid residues with hydrophilic ones.

We wondered whether this contribution of Fab glycans to antibody stability might also apply to naturally acquired Fab glycans in circulating IgGs. Specifically, mutations acquired during somatic hypermutation that improve antigen binding can have destabilizing effects, and additional mutations are introduced to compensate for that (11). Perhaps, acquiring Fab glycans represents a possible mechanism to compensate for those destabilizing mutations. Therefore, we hypothesized that, in addition to their effects on antigen binding, another role of Fab glycans is to improve antibody stability, possibly by shielding hydrophobic residues in the antigen-binding site.

In this study, we demonstrate by analyzing thermal unfolding profiles that introduction of Fab glycans can improve antibody stability. Strikingly, removal of naturally acquired Fab glycans can deteriorate antibody stability, suggesting *in vivo* selection of stable, glycosylated antibodies. Collectively, our data show that Fab glycans can contribute to IgG antibody stability.

## Materials and Methods

### Therapeutic Antibodies

Recombinant therapeutic antibodies used in this study are adalimumab (Humira, Abbvie), cetuximab (Erbitux, Merck), and omalizumab (Xolair, Novartis). Adalimumab is a human monoclonal anti-TNFα IgG1κ antibody, cetuximab is a chimeric monoclonal anti-EGFR IgG1κ antibody, and omalizumab is a humanized monoclonal anti-IgE IgG1κ antibody. Intravenous immunoglobulin (IVIg) was obtained from Sanquin (Nanogam, Amsterdam, The Netherlands) and contains IgGs from thousands of donors.

### Fab Glycovariants

The chimeric monoclonal anti-TNP IgG1κ antibody was produced as described previously (12). The nucleotide sequence of this clone was analyzed for positions where a single nucleotide mutation would suffice to introduce a glycosylation site (hereafter referred to as “progenitor glycosylation site”). A Fab glycovariant with such a glycosylation site effectuated (in the variable domain of the heavy chain) was designed, and a construct coding for the respective variable domain was ordered (Integrated DNA Technologies). The asparagine residue to which a glycan can be attached is located in CDR1 (N_H_29). The human monoclonal anti-TNFα IgG1κ antibody (adalimumab) and adalimumab Fab glycovariants were designed and produced as described previously (3). Briefly, the nucleotide sequence of adalimumab was analyzed for progenitor glycosylation sites. Fab glycovariants with such glycosylation sites effectuated were designed, and constructs coding for the respective variable domains were ordered (Integrated DNA Technologies), 4 with a glycosylation site in the variable domain of the heavy chain and 3 with a glycosylation site in the variable domain of the light chain. The asparagine residues to which glycans can be attached are located in CDR1 (N_L_37), CDR2 (N_H_59), and FR3 (N_H_77, N_H_82, N_H_84, N_L_79, and N_L_86). The human monoclonal patient-derived anti-adalimumab and anti-infliximab antibodies and mutants were obtained, designed, and produced as described previously (3, 13, 14). Briefly, single antigen-specific memory B cells were isolated from patients producing antibodies against adalimumab or infliximab and cultured and screened for specificity. Variable domains were sequenced and analyzed for the presence of glycosylation sites. The asparagine residues to which glycans can be attached are located in CDR1 (anti-infliximab 1.3, N_H_29), FR3 (anti-adalimumab 2.2, N_L_79; anti-adalimumab 2.6, N_H_77; anti-infliximab 2.1, N_H_66), and CDR3 (anti-infliximab 1.4, N_L_110). Constructs coding for anti-adalimumab and anti-infliximab antibodies with naturally acquired glycosylation sites removed (mutated back to germline) were ordered (Integrated DNA Technologies).

Ordered V_H_ and V_L_ sequences in pANY vectors were transformed into DH5α competent cells to amplify DNA. DNA was extracted according to manufacturer’s instructions (NucleoSpin Plasmid EasyPure) and digested out of the pANY vector and ligated/cloned into the pcDNA3.1 expression vector together with the constant domains of the human IgG1 (C_H_) or kappa (C_L_) genes. Next, these sequences were transformed into DH5α competent cells to amplify DNA, and DNA was extracted according to manufacturer’s instructions (NucleoSpin Plasmid EasyPure). Antibodies were expressed by transient cotransfection of heavy and light chain containing vectors into HEK293F cells with either fectin or PEI and Opti-MEM, using the FreeStyle HEK293F expression system according to the instructions supplied by the manufacturer. This expression system reproducibly yields (Fc) glycosylation patterns that closely resemble those found on human serum IgG (15). Cells were incubated for 5 days at 37°C in humidified 8% CO_2_ on a shaker at 125 rpm. Antibodies were purified from culture supernatants at day 5 after transfection using either protein A or protein G Sepharose. Culture supernatants were centrifuged, filtered (0.2 µm), and purified using a HiTrap protein A/G column (GE Healthcare) equilibrated with PBS. IgG was eluted with 0.1 M glycine pH 2.5–3. The fractions were immediately neutralized using 2 M Tris pH 9 and then dialyzed against PBS overnight at 4°C and stored at 4°C.

### Gel Electrophoresis

Samples were analyzed by SDS-PAGE by loading 5 µg IgG on precast 4–12% Bis–Tris gels (NuPAGE), visualized with Coomassie Blue. To examine heavy and light chains separately, samples were reduced with DTT. Figure S1 in Supplementary Material shows original images of gels.

### Lectin ELISAs

Lectin ELISAs were performed as described previously (3). In brief, samples were coated on plates and detected with biotinylated lectin [wheat germ agglutinin (WGA, Vector Laboratories), *Ricinus communis* agglutinin (RCA, Vector Laboratories), or *Sambucus nigra* agglutinin (SNA, Vector Laboratories)], streptavidin labeled with horseradish peroxidase, and tetramethylbenzidine. The reaction was stopped with 2 M H_2_SO_4_, and the absorbance was measured at 450 and 540 nm using a BioTek microtiter plate reader. We previously confirmed that SNA does not bind Fc glycans (3).

### Neuraminidase Treatment

Neuraminidase treatment was performed as described previously (16, 17). Adalimumab N_H_82 and anti-infliximab 1.4 N_L_110 were dialyzed against a 50 mM sodium citrate buffer (pH 6) and incubated for 48 h at 37°C with about 20 U/mL of neuraminidase (20 U/mg IgG) (New England BioLabs). After neuraminidase treatment, samples were dialyzed against PBS and stored at 4°C.

### SNA Lectin Affinity Chromatography

Three different batches of IVIg were fractionated using SNA lectin affinity chromatography as described previously (3) into fractions highly enriched for Fab glycosylation (SA^+^) or depleted for Fab glycosylation (SA^−^). As demonstrated by many studies, SNA binds virtually exclusively to sialic acid residues in Fab glycans due to the relative inaccessibility of Fc glycans in natively folded IgG (18–20). More than 90% of Fab glycans carry terminal sialic acid residues (21).

### Measurement of Antibody Stability

Thermofluor assay analysis was performed using 8-anilino-1-naphthalenesulfonate (ANS) ammonium salt (Fluka), a fluorescent probe that binds to hydrophobic pockets during antibody unfolding (22, 23). This assay was performed analogously to previously described methods (24, 25). Samples of antibodies in PBS containing 60 µM ANS were heated from 45 to 95°C at 1°C/min using a StepOnePlus thermocycler (Thermo Fisher), and fluorescence was measured (excitation by blue diode, emission in channel 1, corresponding to *ca*. 505–535 nm). Initial runs with adalimumab, cetuximab, and omalizumab at concentrations between 0.1 and 10 mg/mL were carried out, and 0.5 mg/mL was chosen as optimal concentration showing clear transitions with fluorescence signals going through a maximum at the expected transition temperatures (*T*_m_); except for anti-adalimumab 2.2, anti-adalimumab 2.6, and anti-infliximab 1.3, where a concentration of 0.5–0.72, 2, and 1 mg/mL was used, respectively. Raw thermograms were smoothed by converting to a 5-point running average, and buffer control runs only containing ANS were subtracted from the data (Figure S2 in Supplementary Material). Reported *T*_m_s are (local) maxima in the fluorescence vs temperature plots, as indicated in Figures S2C, 3B,D, and 4C Supplementary Material.

### Differential Scanning Calorimetry (DSC)

Thermal denaturation was studied using a Nano DSC Calorimeter (TA Instruments) as described previously (26). Samples of the following four antibodies in PBS were diluted to 0.33 mg/mL: adalimumab WT, adalimumab N_H_82, anti-infliximab 1.4 WT, and anti-infliximab 1.4 N110S. After loading the sample into the cell, thermal denaturation was probed from 25 to 90°C at a scan rate of 1°C/min. Buffer correction, normalization, and baseline subtraction procedures were applied, and the data were analyzed using NanoAnalyze Software v.3.7.5 (TA Instruments). The data were fitted using a non-two-state model with two (adalimumab WT, adalimumab N_H_82, anti-infliximab 1.4 WT, and anti-infliximab 1.4 N110S) or three (anti-infliximab 1.4 N110S) peaks and obtained values of *T*_m_ were reported. Reported *T*_m_s are maxima of the 2/3 peaks in the kJ/mol K vs temperature plots. Indicated in Figures 3F and 4H are maxima of the main peak. All samples were measured in two independent DSC runs.

### Statistical Analysis

In all box plots, medians of 3–10 replicates with IQR are shown. To compare two samples, unpaired *t*-tests were done, and to compare multiple samples, one-way ANOVAs compared with WT/total were performed (**P < 0.01, ***P < 0.001, and ****P < 0.0001).

## Results

### Thermostability of an Anti-TNP Clone Carrying Fab Glycans

**Figure 2:**
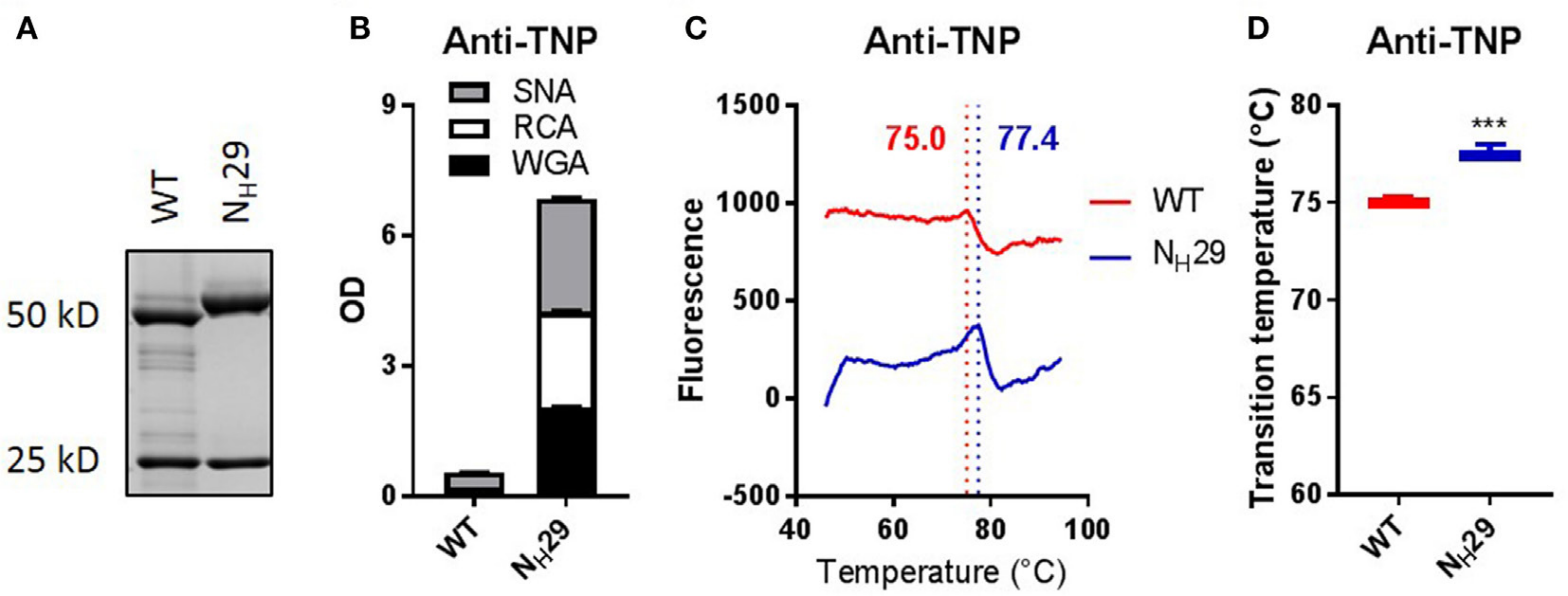
Introducing Fab glycans improves anti-TNP stability. **(A)** Gel electrophoresis of anti-TNP wild-type (WT) and mutant (N_H_29). **(B)** Lectin ELISA data for anti-TNP WT and N_H_29. WGA recognizes *N*-acetylglucosamine, RCA recognizes galactose, and SNA recognizes sialic acid. **(C)** Thermal unfolding profiles of anti-TNP WT (red) and N_H_29 (blue) were determined as described in Figure S3 in Supplementary Material. Thermal unfolding profiles were shifted up or down for clarity. Values in graphs represent obtained *T*_m_ (local maxima). Shown are representative data of three replicates. **(D)** *T*_m_ of anti-TNP variants, determined as described in Figure S3 in Supplementary Material. Shown are medians of three replicates with IQR. Unpaired *t*-test compared with WT, ***P < 0.001.

To evaluate the potential stabilizing effects of variable domain glycans, we first analyzed a chimeric anti-TNP antibody produced in our lab (human IgG1κ; mouse variable domains), and a variant carrying Fab glycans due to introduction of an *N*-linked glycosylation motif. The glycan was introduced at a position designated to be a “progenitor” glycosylation site, meaning that in the context of an antibody immune response, a single nucleotide mutation during somatic hypermutation would suffice to introduce an *N*-linked glycan at this position (3). We confirmed that the anti-TNP variant indeed carried Fab glycans (Figures 2A,B). We noticed that the parent antibody, but not the variant with Fab glycans, has a slight tendency to precipitate. To investigate the thermostability of these clones, we determined thermal unfolding profiles using thermofluor assay analysis with ANS, a fluorescent probe that binds to hydrophobic pockets during protein unfolding (see Materials and Methods). We confirmed that the transition temperatures (*T*_m_) of three therapeutic antibodies (adalimumab, cetuximab, and omalizumab) obtained by this analysis with ANS are in the range of previously published *T*_m_s [Figure S3 and Table S1 in Supplementary Material (25, 27, 28)]. Interestingly, we observed that the anti-TNP variant with Fab glycans had a higher *T*_m_ compared with the antibody without Fab glycans (Figures 2C,D; Table S2 in Supplementary Material), indicating that the variable domain glycans of this clone indeed had a stabilizing effect.

### Introduction of Fab Glycans at Predisposed Locations Can Improve Antibody Stability

**Figure 3:**
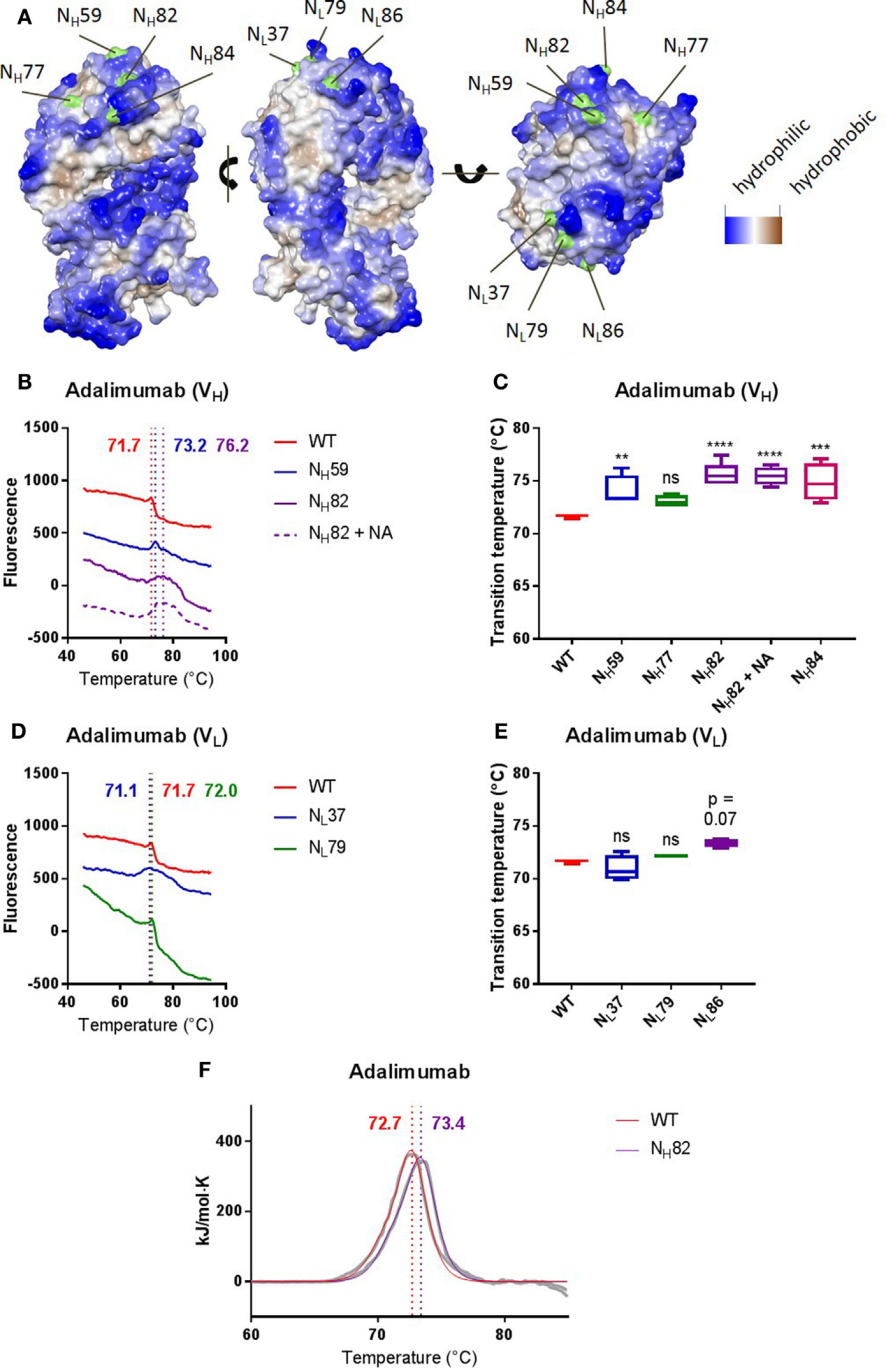
Introducing Fab glycans can improve adalimumab stability. **(A)** The Fab arm of adalimumab with introduced glycosylation sites indicated in green. Sites were introduced in the heavy (left) or light (middle) chain. The more blue, the more hydrophilic, and the more brown, the more hydrophobic. **(B)** Thermal unfolding profiles of adalimumab wild-type (WT, red), V_H_ mutants (N_H_59, blue; N_H_82, purple), and N_H_82 treated with neuraminidase (N_H_82 + NA, dotted purple) were determined as described in Figure S3 in Supplementary Material. Thermal unfolding profiles were shifted up or down for clarity. Values in graphs represent obtained *T*_m_ (local maxima). Shown are representative data of at least four replicates. **(C)** *T*_m_ of adalimumab V_H_ variants, determined as described in Figure S3 in Supplementary Material. Shown are medians of at least four replicates with IQR. One-way ANOVA compared with WT, **P < 0.01, ***P < 0.001, and ****P < 0.0001. **(D)** Thermal unfolding profiles of adalimumab WT (red) and V_L_ mutants (N_L_37, blue; N_L_79, green) were determined as described in Figure S3 in Supplementary Material. Thermal unfolding profiles were shifted up or down for clarity. Values in graphs represent obtained *T*_m_ (local maxima). Shown are representative data of at least four replicates. **(E)** *T*_m_ of adalimumab V_L_ variants, determined as described in Figure S3 in Supplementary Material. Shown are medians of at least four replicates with IQR. One-way ANOVA compared with WT. **(F)** Thermal unfolding profiles of adalimumab WT (red) and N_H_82 (purple) were determined using differential scanning calorimetry. The data are shown in gray and the fits in red and purple. The data were fitted using a non-two-state model with two peaks, and values in graphs represent obtained *T*_m_ (maxima) of the main peak. Shown are representative data of two replicates.

To investigate the effects of Fab glycans on antibody stability in more detail, we next compared the thermostability of adalimumab, a human anti-TNF antibody, with a panel of variants in which we systematically introduced Fab glycosylation sites at several positions across the variable domains corresponding to “progenitor” glycosylation sites as explained earlier. We generated four variants with glycans in the variable domain of the heavy chain (N_H_59, N_H_77, N_H_82, and N_H_84) and three variants with glycans in the variable domain of the light chain (N_L_37, N_L_79, and N_L_86). We previously confirmed that the adalimumab variants indeed carried Fab glycans (3). Figure 1 shows the predicted structure of adalimumab N_H_82. Some introduced glycans were localized close to more hydrophilic residues (N_H_59, N_H_82, N_H_84, and N_L_86), while some glycans were localized close to more hydrophobic residues (N_H_77, N_L_37, and N_L_79) (Figure 3A). We found that several Fab glycovariants had a higher *T*_m_ compared with adalimumab without Fab glycans (N_H_59, N_H_82, and N_H_84, Figures 3B,C; Table S2 in Supplementary Material), which indicates that the Fab glycans introduced in these variants improved antibody stability. For adalimumab variant N_H_82, the stabilizing effects of the Fab glycans were confirmed using DSC (Figure 3F; Table S4 in Supplementary Material). For the other variants, similar thermostabilities were observed with and without Fab glycans. Furthermore, the thermal unfolding profiles of multiple variants, including adalimumab N_L_37, where similar *T*_m_s were observed, did show a broader transition in comparison with adalimumab (Figures 3D,E; Table S2 in Supplementary Material). Interestingly, we did not see this broader peak for all clones with Fab glycans (Table S2 in Supplementary Material). There was no clear correlation between the position of the glycan and the impact on thermostability in terms of proximity to hydrophobic pockets (Figure 3). Our data indicate that sialic acid is not required for the improved stability, since removal of sialic acid by treatment with neuraminidase (Figure S4A in Supplementary Material) did not affect the *T*_m_ for adalimumab N_H_82 (Figures 3B,C; Table S2 in Supplementary Material).

### Removal of Naturally Acquired Fab Glycans Can Deteriorate Antibody Stability

**Figure 4:**
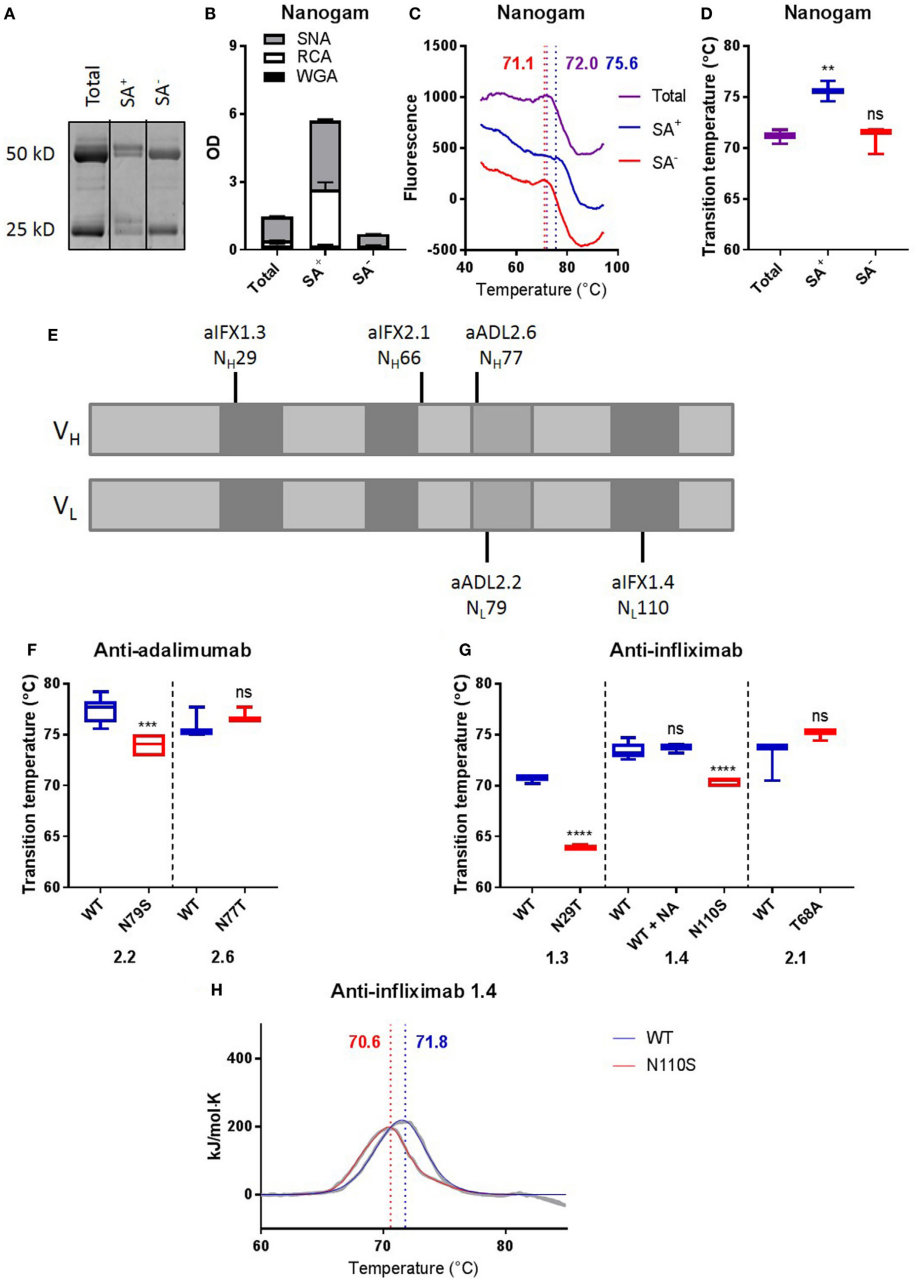
Removing Fab glycans can deteriorate antibody stability. **(A)** Gel electrophoresis of nanogam total, sialic acid enriched (SA^+^), and sialic acid depleted (SA^−^). **(B)** Lectin ELISA data for Nanogam total, SA^+^, and SA^−^. WGA recognizes *N*-acetylglucosamine, RCA recognizes galactose, and SNA recognizes sialic acid. **(C)** Thermal unfolding profiles of Nanogam total (purple), SA^+^ (blue), and SA^−^ (red) were determined as described in Figure S3 in Supplementary Material. Thermal unfolding profiles were shifted up or down for clarity. Values in graphs represent obtained *T*_m_ (local maxima). Shown are representative data of 10 replicates. **(D)** *T*_m_ of Nanogam, determined as described in Figure S3 in Supplementary Material. Shown are medians of three batches with three to four replicates for each batch with IQR. One-way ANOVA compared with total, ****P < 0.0001. **(E)** Positions of glycosylation sites in sequences of two anti-adalimumab and three anti-infliximab clones. Light gray represents framework regions, dark gray represents complementarity determining regions, and middle gray represents DE loop. **(F)** *T*_m_ of anti-adalimumab variants, determined as described in Figure S3 in Supplementary Material. Shown are medians of at least three replicates with IQR. Unpaired *t*-test compared with wild-type (WT), ***P < 0.001. **(G)** *T*_m_s of anti-infliximab variants were determined as described in Figure S3 in Supplementary Material. Shown are medians of at least three replicates with IQR. One-way ANOVA or unpaired *t*-test compared with WT, ****P < 0.0001. **(H)** Thermal unfolding profiles of anti-infliximab 1.4 WT (blue) and mutant (N110S, red) were determined using differential scanning calorimetry. The data are shown in gray and the fits in blue and red. The data were fitted using a non-two-state model with two (WT) or three (N110S) peaks, and values in graphs represent obtained *T*_m_ (maxima) of the main peak. Shown are representative data of two replicates.

To investigate whether Fab glycans naturally acquired during an immune response would also be able to positively contribute to antibody stability, we next compared the thermostability of IVIg enriched for Fab glycans (SA^+^, *ca*. 10%) with that of IVIg depleted for Fab glycans (SA^−^, *ca*. 90%) by SNA lectin affinity chromatography. We confirmed the enrichment and depletion of IVIg for Fab glycans by gel electrophoresis and lectin ELISAs (Figures 4A,B). Interestingly, the (average) *T*_m_ of SA^+^ IVIg (75.6 ± 0.570) was higher than that of SA^−^ IVIg (71.0 ± 0.778), which was similar to that of total IVIg (71.1 ± 0.399) (Figures 4C,D). Relatively broad transitions and high SEMs were observed, which could be explained by the fact that IVIg is a polyclonal antibody preparation. We therefore also investigated the effects of naturally acquired Fab glycans in a number of monoclonal antibodies, with Fab glycans that were acquired *via* natural selection during somatic hypermutation. To this end, we analyzed antigen-specific clones isolated from patients that are treated with adalimumab or infliximab and make antibodies to these biologicals, of which a high proportion carries Fab glycans (3). We compared the thermostability of anti-adalimumab and anti-infliximab antibodies with naturally acquired Fab glycans at different positions [Figure 4E (13, 14)] with mutants in which we removed their Fab glycosylation sites. We previously confirmed that the anti-adalimumab and anti-infliximab mutants indeed did not carry Fab glycans (3). Three out of five tested clones showed a lower *T*_m_ for the mutants without Fab glycans compared with the antibodies with Fab glycans (Figures 4F,G; Table S3 in Supplementary Material), indicating that the naturally acquired Fab glycans in these clones improved antibody stability. For anti-infliximab 1.4, the stabilizing effects of the Fab glycans were confirmed using DSC (Figure 4H; Table S4 in Supplementary Material). Again, sialic acid is not required for this improved stability of anti-infliximab 1.4 (Figures S4B,G and Table S3 in Supplementary Material).

## Discussion

This study is the first to systematically demonstrate that Fab glycans acquired during antigen-specific immune responses may contribute to antibody stability. Our data suggest that improved B-cell receptor (BCR) stability through Fab glycans may contribute to selection of antibodies with Fab glycans *in vivo*. Whether and how stabilizing Fab glycans are indeed selected *in vivo* requires more thorough investigation. It might be possible that Fab glycans affect intracellular stability of BCRs and if a B cell carries a BCR with stabilizing Fab glycans, this B cell might be positively selected. In line with this, affinity-enhancing mutations can sometimes destabilize, which may be counteracted by stability-enhancing mutations (11). The fact that Fab glycans are not present already in naive immunoglobulins but can be introduced at various positions during somatic hypermutation makes this flexible mechanism, subject to the selection mechanisms that ultimately result in the best antigen binders. The alternative, Fab glycans at fixed permanent positions, would not allow a co-evolutionary development of clones that are both optimized for antigen binding and at the same time optimized in terms of stability.

How Fab glycans stabilize antibodies remains to be investigated. It was shown that introduction of glycans specifically introduced to mask aggregation-prone regions improves antibody stability to a similar extent as removing those regions by replacement of hydrophobic amino acid residues with hydrophilic ones (8), suggesting that Fab glycans localized close to hydrophobic residues would shield those. However, for the investigated adalimumab variants, we did not find a correlation between the position of the glycan and the impact on thermostability in terms of proximity to hydrophobic pockets. However, it is difficult to estimate whether attached glycans would be able to shield those hydrophobic residues, since glycans can be very flexible. Negatively charged sialic acid residues might change the overall charge of IgG molecules and thereby stabilize antibodies. However, we show that sialic acid is not required for the improved stability caused by Fab glycans.

Our finding that the introduction of Fab glycans can improve antibody stability is promising for therapeutic as well as diagnostic purposes (facilitation of manufacturing and storage, increase of serum half-life/efficacy, and range of practical applications). However, introduction of Fab glycans to improve antibody stability may affect antigen binding. Nevertheless, we previously showed that for more than one of the generated adalimumab clones with Fab glycans, there were no or relatively small effects of these introduced glycans on antigen binding (3). In particular, the Fab glycans of adalimumab N_H_84 had no effects on antigen binding, whereas they positively contributed to antibody stability, indicating that this could be an interesting drug candidate. For most of the clones where we removed the naturally acquired Fab glycans, we found either no effects or a modestly (twofold) better antigen binding in the presence of Fab glycans.

We determined thermal unfolding profiles using thermofluor assay analysis with ANS, a fluorescent probe that binds to hydrophobic pockets during protein unfolding, and we observed that fluorescence was different for clones with Fab glycans. One could argue that Fab glycans do not necessarily influence antibody stability, but affect ANS binding, or that ANS influences protein unfolding. However, for cetuximab, a therapeutic antibody carrying Fab glycans (27), DSC, a technique without use of a fluorescent probe, provided a *T*_m_ in a similar range as obtained by this analysis with ANS. In addition, for two clones we confirmed the stabilizing effects of the Fab glycans using DSC. For some clones, the position of the peaks was not altered, only their widths. A broader peak and higher SEM might indicate more heterogeneity, and more structural variability through Fab glycans could explain this. Whether such differences in widths of peaks translate in differences in *in vivo* stability is unknown. Also, using DSC, we did not observe such broader peaks.

In conclusion, we demonstrate in this study that variable domain *N*-linked glycans acquired during somatic hypermutation can contribute to IgG antibody stability. Our findings indicate that introducing Fab glycans may represent a mechanism to improve therapeutic/diagnostic antibody stability.

## Author Contributions

TR designed the study; FB, ND, MB, ST, PO-d-H, and RS acquired, analyzed, and interpreted the data; TR supervised the project; FB and TR wrote the manuscript. All coauthors critically reviewed and approved the final manuscript.

## Conflict of Interest Statement

The authors declare that the research was conducted in the absence of any commercial or financial relationships that could be construed as a potential conflict of interest.
