# Supplementary material for: Variable Domain N-Linked Glycans Acquired During Antigen-Specific Immune Responses Can Contribute to Immunoglobulin G Antibody Stability
Source: Front Immunol. 2018 Apr 12;9:740. doi: 10.3389/fimmu.2018.00740 (PMC5906590; doi:10.3389/fimmu.2018.00740)
Supplement: Supplementary file 1 [file Presentation_1.PDF]

Supporting Information

Figure S1. Original images of gels presented in **(A)** Figure 2A and **(B)** 4A.

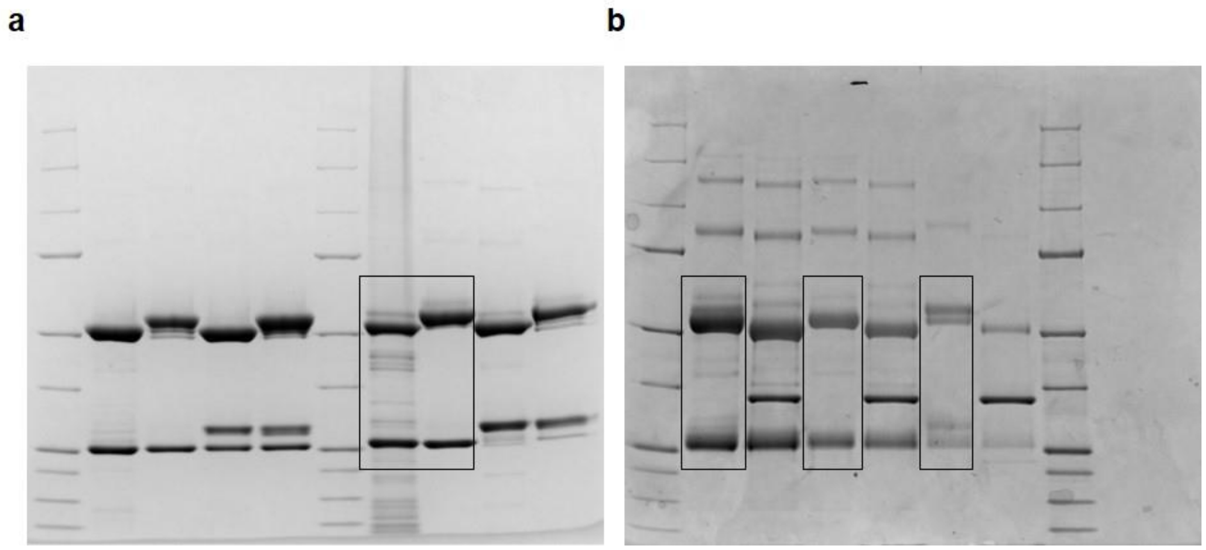

Figure S2. (A) Raw thermograms were **(B)** smoothed by converting to a 5-point running average and **(C)** buffer control runs only containing ANS were subtracted from the data. Thermal unfolding profiles were shifted up or down for clarity.

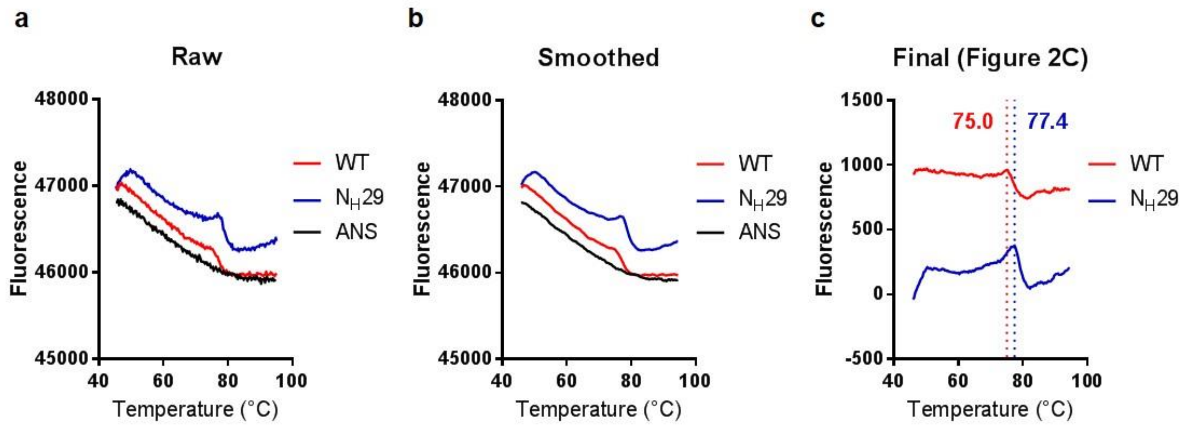

Figure S3. T_ms obtained by analysis with ANS are similar to previously published T_ms. Thermal unfolding profiles of three therapeutic antibodies were determined using thermofluor assay analysis with ANS. Adalimumab (red), cetuximab (blue), and omalizumab (green) were incubated with ANS while heating from 45 °C to 95 °C. Fluorescence was measured, raw thermograms were smoothed and buffer control runs were subtracted. Thermal unfolding profiles were shifted up or down for clarity. Values in graphs represent obtained T_ms (local maxima). Shown are representative data of 3 replicates.

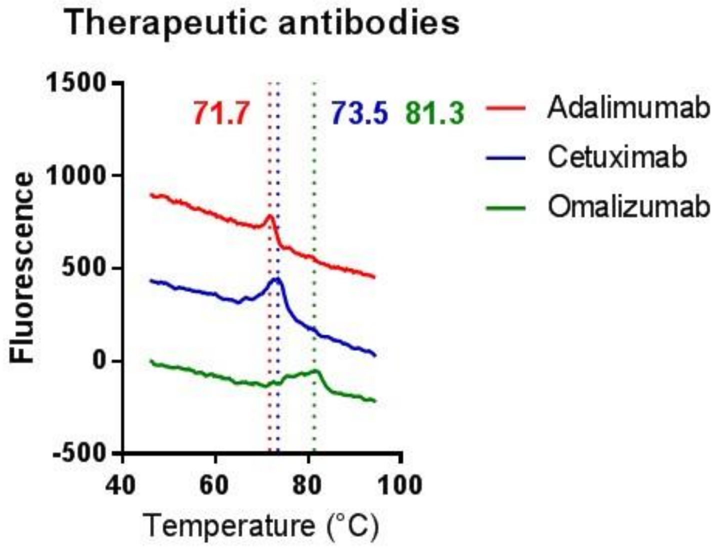

Figure S4. Removal of sialic acid by treatment with neuraminidase. SNA ELISA data for **(A)** adalimumab N_H82 and **(B)** anti-infliximab 1.4 untreated or treated with neuraminidase (+ NA). SNA recognizes sialic acid.

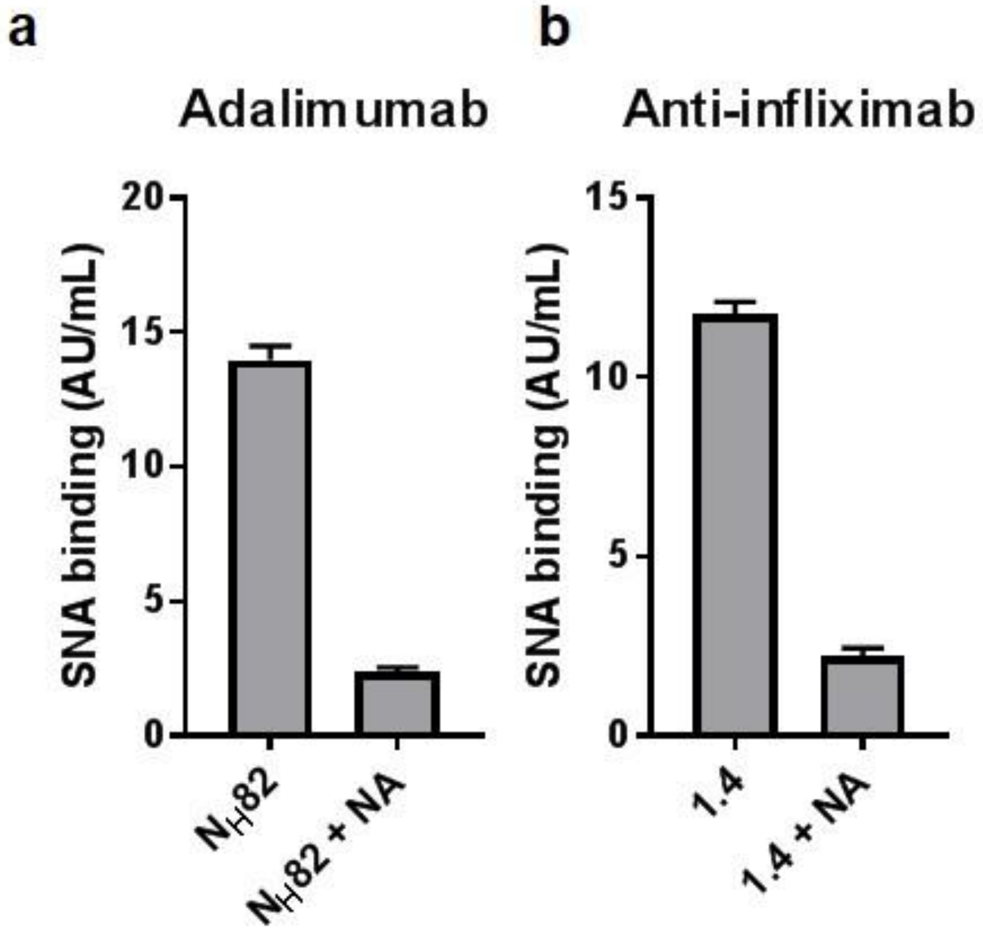

Table S1. Comparison of T_ms found in this study with previously published T_ms of the therapeutic antibodies adalimumab, cetuximab, and omalizumab.

**Antibody clone**	**T_ms**	
	**Found in this study***	**Previously published**
Adalimumab	71.7 ± 0.173	75.0 (24)<br>71.7-72.1 (27)
Cetuximab	73.0 ± 0.361	77.6 (24)<br>~73.0 (26)
Omalizumab	81.6 ± 0.173	83.6 (24)<br>~80.0 (26)

* Mean ± standard error of the mean (SEM) of n = 3 independent measurements with ANS.

Table S2. Overview of antibody clones and corresponding T_ms.

**Antibody clone**	**Mutation**	**Position**	**T_m without Fab glycans***	**T_m with Fab glycans***	**P value**	**Sample size**	**SD****
Anti-TNP	S29N	N_H29 (CDR1)	75.1 ± 0.100 (narrow)	77.6 ± 0.200 (narrow)	0.0004	3 / 3	0.1732 / 0.3464
Adalimumab	G63S	N_H59 (CDR2)	71.7 ± 0.0783 (narrow)	74.0 ± 0.728 (narrow)	0.0044	7 / 4	0.2070 / 1.4570
Adalimumab	T77N	N_H77 (FR3 (DE))	71.7 ± 0.0783 (narrow)	73.1 ± 0.287 (broad)	0.1916	7 / 4	0.2070 / 0.5745
Adalimumab	K84T	N_H82 (FR3 (DE))	71.7 ± 0.0783 (narrow)	75.7 ± 0.420 (broad)<br># 75.5 ± 0.433 (broad)	0.0001<br>0.0001	7 / 6 / 4	0.2070 / 1.0290 / 0.8660
Adalimumab	K84N	N_H84 (FR3 (DE))	71.7 ± 0.0783 (narrow)	74.9 ± 0.896 (broad)	0.0001	7 / 4	0.2070 / 1.7920
Adalimumab	L39S	N_L37 (CDR1)	71.7 ± 0.0783 (narrow)	71.0 ± 0.606 (broad)	0.6851	7 / 4	0.2070 / 1.2120
Adalimumab	S79N	N_L79 (FR3 (DE))	71.7 ± 0.0783 (narrow)	72.2 ± 0.0866 (narrow)	0.9844	7 / 4	0.2070 / 0.1732
Adalimumab	D86N	N_L86 (FR3 (DE))	71.7 ± 0.0783 (narrow)	73.4 ± 0.194 (narrow)	0.0694	7 / 4	0.2070 / 0.3873

* Mean ± standard error of the mean (SEM) of n = 3-6 independent measurements with ANS.

** Standard deviation (SD)

Neuraminidase treated

Table S3. Overview of antibody clones and corresponding T_ms.

**Antibody clone**	**Position**	**Mutation**	**T_m with Fab glycans***	**T_m without Fab glycans***	**P value**	**Sample size**	**SD****
Anti-adalimumab 2.2	N_L79 (FR3 (DE))	N79S	77.3 ± 0.480 (broad)	74.1 ± 0.359 (narrow)	0.0002	7 / 7	1.2690 / 0.9487
Anti-adalimumab 2.6	N_H77 (FR3 (DE))	N77T	76.0 ± 0.854 (narrow)	76.9 ± 0.400 (narrow)	0.3941	3 / 3	1.4800 / 0.6928
Anti-infliximab 1.3	N_H29 (CDR1)	N29T	70.6 ± 0.200 (broad)	64.0 ± 0.100 (narrow)	< 0.0001	3 / 3	0.3464 / 0.1732
Anti-infliximab 1.4	N_L110 (CDR3)	N110S	73.4 ± 0.363 (narrow)<br># 73.7 ± 0.265 (narrow)	70.4 ± 0.204 (narrow)	0.0001<br><br>0.7126	5 / 3 / 5	0.8106 / 0.4583 / 0.4550
Anti-infliximab 2.1	N_H66 (FR3)	T68A	72.7 ± 1.10 (broad)	75.0 ± 0.300 (narrow)	0.1139	3 / 3	1.9050 / 0.5196

* Mean ± standard error of the mean (SEM) of n = 3-5 independent measurements with ANS.

** Standard deviation (SD)

Neuraminidase treated

Table S4. Overview of antibody clones and corresponding T_ms obtained by DSC.

**Antibody clone**	**T_m with Fab glycans***	**T_m without Fab glycans***	**P value**	**Sample size**	**SD****
Adalimumab (N_H82)	T_m1 71.55 ± 0.330<br>T_m2 73.43 ± 0.015	T_m1 70.54 ± 0.125<br>T_m2 72.66 ± 0.035	0.1026<br>0.0025	2 / 2	0.4667 / 0.1768<br>0.0212 / 0.0495
Anti-infliximab 1.4#	T_m1 69.84 ± 0.165<br>T_m2 71.85 ± 0.020	T_m1 69.23 ± 0.020<br>T_m2 70.92 ± 0.005	0.0679<br>0.0005	2 / 2	0.2333 / 0.0283<br>0.0283 / 0.0071

* Mean ± standard error of the mean (SEM) of n = 2 independent measurements with DSC.

** Standard deviation (SD)

Obtained T_ms for anti-infliximab 1.4 without Fab glycans (N110S) fitted with three peaks (that fit is presented in **Figure 4H**) were 68.32 ± 0.015 (P value = 0.0117), 70.60 ± 0.015 (P value = 0.0004), and 74.05 ± 0.060.
